# Multivariable Linear Algebraic Discretization of Nonlinear Parabolic Equations for Computational Analysis

**DOI:** 10.1155/2022/6323418

**Published:** 2022-09-29

**Authors:** Li Zuo, Fengtai Mei

**Affiliations:** ^1^School of Nursing, Chengdu Polytechnic, Chengdu 610071, China; ^2^College of General Education, Chengdu Polytechnic, Chengdu 610071, China

## Abstract

Since the nonlinear parabolic equation has many variables, its calculation process is mostly an algebraic operation, which makes it difficult to express the discrete process concisely, which makes it difficult to effectively solve the two grid algorithm problems and the convergence problem of reaction diffusion. The extended mixed finite element method is a common method for solving reaction-diffusion equations. By introducing intermediate variables, the discretized algebraic equations have great nonlinearity. To address this issue, the paper proposes a multivariable linear algebraic discretization method for NPEs. First, the NPE is discretized, the algebraic form of the nonlinear equation is transformed into the vector form, and the rough set (RS) and information entropy (IE) are constructed to allocate the weights of different variable attributes. According to the given variable attribute weight, the multiple variables in the equation are discretized by linear algebra. It can effectively solve the two grid algorithm problems and the convergence problem of reaction diffusion and has good adaptability in this field.

## 1. Introduction

When the relationship between the two variables is not linear, it is said to be nonlinear parabolic. Some of the relations could be square, logarithmic, exponential, trigonometric function relationships, etc. Because a parabolic equation can describe the state or process of a physical quantity changing with time, it has many applications in real life, such as heat conduction, liquid permeation, and gas permeation. For example, the heat conduction NPE is a differential equation that describes the dispersion of atmospheric pollutant concentration, coastal salinity, and the law of fluid motion, while the delay NPE is also widely used, such as in the fields of population dynamics, ecology, and environmental science. The data simulation problems can be reduced to a delay NPE [1]. There are many variables in the NPE. When the NPE is used in practice, the amount of data corresponding to each variable is huge. When the number of variables in the NPE increases, it becomes very difficult to solve the equation. Therefore, it is necessary to discretize the variables of NPE.

At present, some scholars have studied it. For example, document [2] proposes a three-step, two-layer grid method for the nonlinear reaction-diffusion problem, that is, solving the original nonlinear algebraic equations on the coarse grid (CG) and then correcting the CG . Through convergence analysis, it is found that the three-step two-layer mesh method can maintain the asymptotic optimal approximation of the mixed finite element solution when the selected coarse space step size is satisfied. However, this method has high computational complexity and low efficiency. When the variable data distribution is non-Gaussian, the second-order statistics method that relies on the data is often ineffective. It does not explicitly give the number of principal components, which may affect the results of discretization. In addition, some traditional discretization methods are mostly aimed at a single variable, and the calculation process is mostly an algebraic operation, making it difficult to express the discrete process concisely [3]. Therefore, to solve the two grid algorithm problems of NPE and the convergence problem of reaction diffusion, this paper will study the multivariable linear algebraic discretization method of NPE to provide some help for solving the multivariable linear algebraic discretization problem of NPE.

## 2. Multivariable Linear Algebraic Discrete Methods for NPEs

### 2.1. Discretization of NPEs

For the NPE, the discretization of variables is used to discretize the attributes of the variables to solve the NPE. Discretization is the process of mapping a finite number of individuals in an infinite space into a finite space to improve the spatial and temporal efficacy of data or variables. Discretization is the process of reducing the size of data without making changes in the relative size of the variable data. Discretization essentially comes down to the problem of dividing the space of conditional attributes by using the selected breakpoints. The space of attributes is divided into a finite area, so that the decision values of objects in each area are the same. If an attribute has an attribute value, then there is a breakpoint on the attribute that is desirable. As the number of attributes increases, the number of desirable breakpoints increases geometrically. The process of selecting breakpoints is also the process of merging attribute values [4]. By merging attribute values, the number of attribute values and the number of breakpoints can be reduced, and thus the complexity of the problem can be reduced. The current discretization methods are all aimed at single variables, and they consider only one attribute for discrete attributes. Therefore, the result of the discretization of a single variable is often not optimal because the target class in variable data is determined by multiple attributes rather than by a single attribute.

To discretize the NPE, the scheme of the NPE should be changed. The discrete expression of the NPE is as follows:

(1) $$\begin{array}{c} \left\{\begin{array}{c} u_{t}-u_{xx}-\frac{\sigma }{x}u=f\left(u\right),\left(x,t\right)\in \Omega =\left(0,1\right)\times \left(0,T\right)\text{,}\\ u_{x}\left(0,t\right)=u\left(1,t\right)=0,t\in \left(0,T\right)\text{,}\\ u\left(x,0\right)=0,x\in \Gamma \text{,}\\ \Gamma =\left\{x:x\in \left(0,1\right)\right\}\text{.} \end{array}\right. \end{array}$$

In the above formula (1), the nonhomogeneous term *f*(*u*) at the right end of the equation satisfies continuity, that is, for ∀*v*, *ω* ∈ *R*, there is a constant *L*, which makes |*f*(*x*, *t*, *v*) − *f*(*x*, *t*, *ω*)| ≤ *L*|*v* − *ω*| hold. Take the space step *h*=1/*M*, the time step *k*=1/*N* (*M*, *N* are positive integers), and note that the space node is *x*_*m*_=*mh*, the time node is *t*_*n*_=*nk*, and the numerical solution is *un*/*m* ≈ *u*(*x*_*m*_, *t*_*n*_). If the solution *u* of formula (1) has the necessary differentiability, then it is as follows:

(2) $$\begin{array}{c} u\left(x_{m+1},t\right)-2u\left(x_{m},t\right)+u\left(x_{m-1},t\right)=\frac{\partial^{2}u\left(x_{m},t\right)}{\partial x^{2}}h^{2}+\frac{1}{12}\frac{\partial^{4}u\left(x_{m},t\right)}{\partial x^{4}}h^{4}+o\left(h^{6}\right)\text{.} \end{array}$$

After a series of simplification and consolidation operations on formula (2), according to the relationship existing in formula (1).

(3) $$\begin{array}{c} \frac{\partial^{2}u\left(x,t\right)}{\partial x^{2}}=\frac{\partial u\left(x,t\right)}{\partial t}-f\left(x,t\right)\text{.} \end{array}$$

From the Taylor expansion (3), we can get the following difference transformation scheme for NPE.

(4) $$\begin{array}{c} \frac{k}{h^{2}}\left[u\left(x_{m+1},t\right)-2u\left(x_{m},t\right)\right]+u\left(x_{m-1},t\right)\\ =\frac{k}{12}\frac{\partial u\left(x_{m+1},t\right)}{\partial t}+\frac{10k}{12}\frac{\partial u\left(x_{m},t\right)}{\partial t}+\frac{k}{12}\frac{\partial u\left(x_{m-1},t\right)}{\partial t}\\ -\frac{k}{12}f\left(x_{m+1},t\right)-\frac{10k}{12}f\left(x_{m},t\right)-\frac{k}{12}f\left(x_{m-1},t\right)+o\left(k^{2}+kh^{4}\right)\text{.} \end{array}$$

To make a linear algebraic discretization of the transformed NPE, the difference scheme shown in the above formula is transformed into the vector form shown in the following formula.

(5) $$\begin{array}{c} rAu^{n}+Bu^{n}=Bu^{n-1}+kBf^{n-1}+d^{n}\text{.} \end{array}$$

In the above formula (5), *r*, *A*, and *B* are all tridiagonal matrices of order (*M* − 1) × (*M* − 1). *u*^*n*^, *f*^*n*−1^, and *d*^*n*^ are all *M* − 1-dimensional column vectors, and *k* is the Taylor expansion order. The parameters of matrix form of NPE are as follows:

(6) $$\begin{array}{c} \left\{\begin{array}{c} r=\frac{k}{h^{2}}\text{,}\\ A=tri\;di\;ag\left(-1,2,-1\right)\text{,}\\ B=tir\;di\;ag\left(\frac{1}{12},\frac{10}{12},\frac{1}{12}\right)\text{.} \end{array}\right. \end{array}$$

After transforming the NPE into vector form, several variables in the equation are treated with linear algebraic discretization [5–7]. The RS and IE of NPE variables are constructed.

### 2.2. Construction of RSs and IE

To deal with imprecise data, RS is used. RSs do not need any prior knowledge or additional information about the data. In RS, a variable data table is called an information system (IS). The IS is a quad *G*_*IS*_=(*U*, *W*, *V*, *f*), which meets the following four conditions:

1. *U* represents a nonempty set of objects.
2. *W* represents a nonempty finite attribute set;
3. *V* is the set of attribute values, *V*=*U*(*V*_*w*_), *V*_*w*_ is the value field of attribute *w*.
4. *f*: *U* × *W*⟶*V* is a mapping function, which represents a value of the attribute value set mapped by each attribute of each object;

If an attribute from the attribute set is considered a decision attribute, IS *G*_*IS*_ is also called decision-table. Expressed as: *T*_*D*_=(*U*, *C*, *D*, *V*, *f*), among them, *C* ∪ *D*=*W*, *C*∩*D*=*ϕ*; *C* is called the set of conditional attributes; *D* is the set of decision-attributes. In a given decision-table (DT), there is an indistinguishable relationship; that is, there is a certain interaction between the two variables. Indistinguishable relations are defined as follows:

for a given DT *T*_*D*_=(*U*, *C*, *D*, *V*, *f*) and *Q* ∈ *C* ∪ *D*, the binary relation *IND*(*Q*) is called indistinguishable relation. The definition formula is as follows:

(7) $$\begin{array}{c} IND\left(Q\right)=\left\{\left(x,y\right)\in U\times U\right\}\text{.} \end{array}$$

The above formula (7) satisfies ∀*a* ∈ *Q*(*f*(*x*, *a*)=*f*(*y*, *a*)). Indistinguishable relation *IND*(*Q*) is equivalent relation on *U*, which divides *U* into disjoint equivalent classes. *U*/*IND*(*Q*) represents the set of equivalence classes of an equivalence relation *IND*(*Q*) on *U*, *U*/*Q* for each object *x* ∈ *U*, the equivalent class of element *x* in *U*/*Q* is represented by [*x*]*Q*. It is called the equivalence class of object *x* on equivalence relation *IND*(*Q*). Using the new DT instead of the original one can reduce the variable attributes of the NPE. After constructing the RS, the IE of the NPE is calculated.

IE is used to describe the purity of data sets. When data sets belong to a certain category, the IE is 0. When the data of data sets are more mixed, the IE is higher. IE and discrete breakpoint IE are defined as follows:

(8) $$\begin{array}{c} \left\{\begin{array}{c} E\left(S\right)=-\overset{s}{\underset{i=1}{\sum }}P\left(C_{i},S\right)\log\;\left(P\left(C_{i},S\right)\right)\text{,}\\ E\left(W,T,S\right)=\frac{\left|S_{1}\right|}{\left|S\right|}Ent\left(S_{1}\right)+\frac{\left|S_{2}\right|}{\left|S\right|}Ent\left(S_{2}\right)\text{.} \end{array}\right. \end{array}$$

In the above formula (8), *S* is the set of objects; *s* is the number of variables in the NPE; *C*_*i*_ represents the number of variables of type *i* in object set *S*; *W*, *T* represent breakpoint *T* on attribute *W*, respectively. |*S*| is the cardinality of set *S* [8–10]. After constructing the RS and IE of the NPE, the breakpoint is selected and the discrete decision tree is established.

### 2.3. Model Problem

Through the reaction diffusion problem of the mathematical model of the porous medium groundwater flow problem, and explain the physical meaning of the parameters in the model [11–13]. This model can be described by a set of nonlinear partial differential equations. The nonlinear reaction diffusion equation is given as follows:

(9) $$\begin{array}{c} \frac{\partial p}{\partial t}-\nabla \times \left(K\left(P\right)\nabla p\right)=f\left(p,\nabla p\right),\left(x,t\right)\in \Omega \times J\text{.} \end{array}$$

The initial conditions are given as follows:

(10) $$\begin{array}{c} p\left(x,0\right)=p^{0}\left(x\right),x\in \Omega \text{.} \end{array}$$

The boundary conditions are given as follows:

(11) $$\begin{array}{c} K\left(P\right)\nabla p\times v=0,\left(x,t\right)\in \partial \Omega \times J\text{,} \end{array}$$

where Ω ⊂ *R*^2^, is the polygonal region whose boundary is marked as *∂*Ω [14, 15]. Where *v* is the normal vector outside the unit of *∂*Ω, *J*=(0, T] and *K* are the tensor of the square integrable symmetric positive definite. It is composed of the first order conservation of mass and the following equations with respect to energy *P* and relative velocity *u*.

(12) $$\begin{array}{c} \frac{\partial p}{\partial t}+\nabla \times u=f\left(p,\nabla p\right)\text{,} \end{array}$$

(13) $$\begin{array}{c} K{\left(P\right)}^{-1}u+\nabla p=0\text{,} \end{array}$$

where the strain in the equations (12) and (13) is *p* and *u*. *p* is the unknown fluid pressure; *u* is the flow rate of the liquid; and *f*(*p*, ∇*p*) is the external flow rate [16–18]. Firstly, the weak form of the equation and the fully discrete scheme of the extended mixed finite element are established. Then, the error estimates of the fully discrete scheme are obtained by using the projection operator and its approximation properties.

Let the following three variables, pressure *p*, gradient *τ*=∇*p*, and flow *φ*=*K*(*p*)*τ*. The weak form of the initial boundary value problem is defined, i e. there is $\left(p,\tau ,\varphi \right)\in W\times V\times \tilde{V}$, so that,

(14) $$\begin{array}{c} \left(\frac{\partial p}{\partial t},\omega \right)+\left(\nabla \times \varphi ,\omega \right)=\left(f\left(p,\tau \right),\omega \right),\omega \in W\text{,} \end{array}$$

(15) $$\begin{array}{c} \left(\tau ,v\right)=-\left(p,\nabla \times v\right),v\in \tilde{V}\text{,} \end{array}$$

(16) $$\begin{array}{c} \left(\varphi ,v\right)=\left(K\left(p\right)\tau ,v\right),v\in V\text{,} \end{array}$$

according to the weak form (14)–(16) of the initial boundary value problem, *RT*_*k*_ group of time discrete extended mixed finite element approximations can be established on the a-element, given $\left(pn/h,\tau n/h,\varphi n/h\right)\in W_{h}\times V_{h}\times \tilde{V_{h}}$, for *n*, *n*=1, Λ, *N*, let $\left(pn/h,\tau n/h,\varphi n/h\right)\in W_{h}\times V_{h}\times \tilde{V_{h}}$ satisfy the following formula:

(17) $$\begin{array}{c} \left(\frac{p_{h}^{n}-p_{h}^{n-1}}{t},\omega_{h}\right)+\left(\nabla \times \varphi_{h}^{n},\omega_{h}\right)=\left(f\left(p_{h}^{n},\tau_{h}^{n}\right)\right),\omega_{h}\in W_{h}\text{,} \end{array}$$

(18) $$\begin{array}{c} \left(\tau_{h}^{n},v_{h}^{n}\right)=-\left(p_{h}^{n},\nabla \times v_{h}\right),v_{h}\in \tilde{V_{h}}\text{,} \end{array}$$

(19) $$\begin{array}{c} \left(\varphi_{h}^{n},v_{h}\right)=\left(K\left(p_{h}^{n}\right)\tau_{h}^{n},v_{h}\right),v_{h}\in V_{h}\text{.} \end{array}$$

The existence and uniqueness of the solutions of the nonlinear equations (17)–(19) have been proved.

At the same time, the minimum number of breakpoints is obtained and the indiscernible relationship between objects is ensured. The reasonable criteria for breakpoint selection are generally: consistency, irreducibility, and minimum discreteness. Given a DT *T*_*D*_=(*U*, *C*, *D*, *V*, *f*), and *C*_*i*_ is the candidate breakpoint (CBP) set of the *i* conditional attribute *a*_*i*_ ∈ *C* on the domain *U*. *C*_*s*_ is a subset of *U*, *C*_*si*_ is the CBP set of the *i*-th conditional attribute *a*_*i*_ ∈ *C* on the domain *C*_*s*_, where *C*_*si*_ is a subset of *C*_*i*_.

In this paper, the label {*a*_*i*_|*C*_*s*_} is used to represent the set of value fields of attribute *a*_*i*_ on a subdomain *U*_*s*_. The formalized formula is as follows:

(20) $$\begin{array}{c} C_{si}=\left\{c\left|c\in C_{i}\;\wedge \;\min\;\left(a_{i}\left|C_{s}\right.\right)<c<\;\max\;\left(a_{i}\left|C_{s}\right.\right)\right.\right\}\text{.} \end{array}$$

The specific treatment process is given as follows:

1. If the DT contains numerical attributes, the algorithm afc4.5 is used to discretize them.
2. According to the processed data set *U*, the discernibility matrix is generated, and the frequency function value of each attribute C is calculated, and the result is taken as the importance measure of the decision attribute *D*.
3. Prepruning of decision the tree: calculate the frequency function value and sum *s* of each attribute, and get the attribute *a* with the largest value and its corresponding value *r*, if *r*/*s* is greater than a certain threshold, it reaches the leaf node. Otherwise, a is taken as the current test attribute.
4. Based on *a*, a discrete decision tree is constructed, *Root* ← *r* (Root is the root node), then test each possible value *v*_*i*_ of *r*. Add a new branch corresponding to test *r*.value=*v*_*i*_ under *Root*⟵*r*. *X*_*v*_*i*__ is the subtable of *U* that satisfies the attribute value of *r* as *v*_*i*_. For each subtable *X*_*v*_*i*__, if the leaf node is not reached, *AFC*4.5(*X*_*v*_*i*__, *W* − {*a*}) is called recursively.

The value of each matrix element in the judgment matrix (JM), i.e., the importance of different Table 1 attributes of the same NPE variable, can be determined according to the following table:

According to the knowledge of linear algebra, the maximum eigenvalue of JM *R*=(*r*_*mn*_)_*ij*_ and its corresponding eigenvector are calculated as follows:

(21) $$\begin{array}{c} R=\lambda_{\max}\times w*\text{.} \end{array}$$

In the above formula (21), *λ*_max_ is the maximum eigenvalue of JM *R*=(*r*_*mn*_)_*ij*_, and its corresponding eigenvector *w∗*=(*w*_1_*∗*, *w*_2_*∗*, ⋯, *w*_*n*_*∗*). When experts compare the properties of NPE in two, it is impossible to achieve the same measurement, and there will be some errors. Therefore, to improve the reliability of determining the weight value, it is necessary to check the consistency of the JM.

When the JM *R*=(*r*_*mn*_)_*ij*_ is completely consistent, *λ*_max_=*n*. However, in general, it is difficult to achieve. To test the consistency of the JM, the following formula is needed to calculate its consistency index *CI*:

(22) $$\begin{array}{c} CI=\frac{\left|\lambda_{\max}-n\right|}{n-1}\text{.} \end{array}$$

In the above formula (22), when *CI* = 0, the JM *R*=(*r*_*mn*_)_*ij*_ has complete consistency. On the contrary, the larger *CI* is, the less consistent the JM *R*=(*r*_*mn*_)_*ij*_ is. To test whether JM *R*=(*r*_*mn*_)_*ij*_ has satisfactory consistency, it is necessary to compare *CI* with an average random consistency index *RI* to get *CR*, that is,

(23) $$\begin{array}{c} CR=\frac{CI}{RI}\text{.} \end{array}$$

The average random consistency index *RI* is shown in Table 2.

When *CR* < 0.1, the JM *R*=(*r*_*mn*_)_*ij*_ has satisfactory consistency; when *CR* ≥ 0.1, adjust the JM *R*=(*r*_*mn*_)_*ij*_ until it is satisfied [19]. At this time, the attribute weights of the variables of the NPE are normalized to get the attribute weights of the variables of the NPE. After the attribute weights of the variables of the NPE are determined, the linear algebraic discretization of the variables of the equation is completed.

### 2.4. Realize Linear Algebraic Discretization of Variables

The linear algebraic discretization of a multivariable nonlinear parabolic method is to discretize the whole variable data set. Its basic idea is to fully consider the overall distribution of variables in the attribute space composed of all variable attributes and to use the complementarity and correlation of different attributes in distinguishing objects to generalize the discretization of the variable attribute space of the equation.

Multivariable linear algebraic discretization of NPEs is used to obtain the CBP set. The CBP set is assumed to be empty, and the CBP set for each continuous attribute is added to the CBP set. CBP sets for multivariable linear algebraic discretization include CBPs for all continuous attributes in the data set. After the initial breakpoint set is obtained, the optimal breakpoint is found. The optimal breakpoint is looked up using the objective function. The breakpoint selection of multivariate linear algebraic discretization is the CBP of all variable attributes, considering the complementarity and correlation of variable attributes. After deleting or adding a breakpoint to get the optimal breakpoint, the optimal breakpoint is put into the optimal breakpoint set, and the optimal breakpoint set is initially empty, and the breakpoint is deleted from the initial breakpoint set. In this case, the optimal breakpoint is the best breakpoint for partitioning continuous attributes, and the final breakpoint required for a dataset is in the optimal breakpoint set. In multivariable discretization, the splitting method first finds the optimal breakpoint from all the continuous attributes and then splits the objects in the dataset.

The first step is to find the initial breakpoint. The second step is to find the best breakpoint, and then to divide the continuous attribute values according to the breakpoint.

According to the results of attribute splitting, the variables are merged. In this paper, the method of clustering is used to merge the NPEs. The central idea of grid-based clustering is to give a large set of multivariate data points, which are generally unevenly distributed in the data space. Multivariable linear algebraic discretization is equivalent to the process of hypercube partitioning of variable feature space by hyperplanes perpendicular to different continuous attribute axes. The discrete partition points on each continuous attribute axis correspond to the intersection points of the attribute axis and the hyperplanes divided perpendicular to it. Multivariate linear algebraic discretization is a process in which hyperplanes are determined independently on each continuous attribute axis. The number of hyperplane partitions and the importance of variable attributes in NPE obey certain probability distributions. According to the mathematical probability principle, we can determine the number of hyperplanes in a linear algebraic discretization of different NPEs and use hyperplanes to discretize the variables in the attribute space. Thus, the multivariable linear algebraic discrete method for NPEs is studied.

## 3. Multivariable Linear Algebraic Discrete Method and its Convergence Analysis

### 3.1. Multivariable Linear Algebraic Discrete Method

The two new two-layer mesh methods and their convergence analysis for the extended hybrid finite element method for nonlinear parabolic discretization problems. By using the idea of correcting on CG, some new two-layer grid methods can be obtained. From the analysis of convergence of the discrete method, it is found that this method is obviously more effective than the existing two-layer grid method. Applying the idea of a Newton iteration on fine meshes and correction on coarse meshes to the extended hybrid finite element method of nonlinear reaction diffusion problems, a two-layer mesh algorithm is constructed.

The solution of a system of nonlinear equations in a fine space is decomposed into a system of nonlinear equations in a rough space, and then a Newtonian iterative system of linear equations in a fine space, and then a system of linear equations in a CG as a correction. This method can be divided into the following three steps:

Step 1: On the CG *F*_*H*_, for any *w*_*H*_ ∈ *W*_*H*_, $v_{H}\in {\tilde{V}}_{H}$, *v*_*H*_ ∈ *V*_*H*_, $\left(p_{H}^{n}-t_{H}^{n},w_{H}^{n}\right)\in W_{H}\times V_{H}\times {\tilde{V}}_{H}$ is calculated to satisfy the following nonlinear equations:
  (24) $$\begin{array}{c} \left(\frac{\left(p_{H}^{n}-p_{H}^{n-1},w_{H}\right)}{\Delta t},w_{H}\right)+\left(\nabla \times \varphi_{H}^{n},w_{H}\right)=\left(f\left(p_{H}^{n}\right),w_{H}\right)\left(t_{H}^{n},v_{H}\right)=\left(p_{H}^{n},\nabla \times v_{H}\right)\left(\varphi_{H}^{n},v_{H}\right)=\left(K\left(p_{H}^{n}\right)t_{H}^{n},v_{H}\right)\text{.} \end{array}$$
Step 2: On the CG *F*_*h*_, for any *w*_*h*_ ∈ *W*_*h*_, $v_{h}\in \tilde{V_{h}}$, *v*_*h*_ ∈ *V*_*h*_, $\left(\tilde{p}n/h-\tilde{t}n/h,\tilde{w}n/h\right)\in W_{h}\times V_{h}\times {\tilde{V}}_{h}$ is calculated to satisfy the following nonlinear equations:
  (25) $$\begin{array}{c} \left(\frac{\left({\tilde{p}}_{H}^{n}-{\tilde{p}}_{H}^{n-1},w_{H}\right)}{\Delta t},w_{h}\right)+\left(\nabla \times {\tilde{\varphi }}_{H}^{n},w_{H}\right)=\left(f\left(p_{H}^{n}\right)+f_{p}\left(p_{H}^{n}\right),w_{h}\right)\left({\tilde{t}}_{h}^{n},v_{h}\right)=\left({\tilde{p}}_{h}^{n},\nabla \times v_{h}\right)\left({\tilde{\varphi }}_{h}^{n},v_{h}\right)=\left(K\left(p_{H}^{n}\right)t_{h}^{n},v_{h}\right)+\left(K\left(p_{H}^{n}\right)t_{h}^{n},v_{h}\right)\text{.} \end{array}$$
Step 3: On the CG *F*_*H*_, for any *w*_*H*_ ∈ *W*_*H*_, $v_{H}\in {\tilde{V}}_{H}$, *v*_*H*_ ∈ *V*_*H*_, $\left({\bar{p}}_{H}^{n}-{\bar{t}}_{H}^{n},{\bar{w}}_{H}^{n}\right)\in W_{H}\times V_{H}\times {\tilde{V}}_{H}$ is calculated to satisfy the following nonlinear equations:
  (26) $$\begin{array}{c} \left(\frac{{\bar{p}}_{H}^{n}-{\bar{p}}_{H}^{n-1},{\bar{w}}_{H}}{\Delta t},w_{H}\right)+\left(\nabla \times {\bar{\varphi }}_{H}^{n},w_{H}\right)=\left(f\left({\bar{p}}_{H}^{n}\right),w_{H}\right)\left({\bar{t}}_{H}^{n},v_{H}\right)=\left({\bar{p}}_{H}^{n},\nabla \times v_{H}\right)\left({\bar{\varphi }}_{H}^{n},v_{H}\right)=\left(T_{1},v_{H}\right)\text{.} \end{array}$$

Among them,

(27) $$\begin{array}{c} T_{1}=K\left(p_{H}^{n}\right){\bar{t}}_{H}^{n}+K_{p}\left(p_{H}^{n}\right){\bar{p}}_{H}^{n}t_{H}^{n}+K_{p}\left(p_{H}^{n}\right)\left({\bar{p}}_{h}^{n}-p_{H}^{n}\right)\left({\bar{t}}_{h}^{n}-t_{H}^{n}\right)\text{.} \end{array}$$

Several lemmas need to be quoted again to complete the above proof.

### 3.2. Lemmas

Symmetric lemma, the integer *s* is the central axis of symmetry of *u*(!*s*)/*i*, and the number of *u*(!*s*)/*i* is equal in the range of equal distance on both sides of the axis of symmetry.

Corresponding lemma if *s*, *t*, *m*, *k*are integers and *s* − *t*=*m*, then the number of *u*(!*t*)/*i* in the interval (*t*, *t*+*k*) is equal to the number of *u*(!*t*)/*i* in the interval (*s*, *s*+*k*), that is, the number of two corresponding combinations is equal in the corresponding interval of equal length [1].

**Lemma 1 .**

$\underset{i⟶\infty }{\lim p_{i}/\omega_{i,v}^{3}}=\infty$ , $\lim_{i⟶\infty }p_{i}/\omega_{i,v}^{2}=\infty$$\lim_{i⟶\infty }p_{i}/\omega_{i,v}=\infty$.

Prove:

(1) ∵*ω*_*i*,*v*_=*A*_*i*_/*B*_*i*_
Set up *a*_*i*_=*p*_*i*_/*ω*_*i*,*v*_^3^=(*B*_*i*_/*A*_*i*_)^3^*p*_*i*_ , *a*_*i*+1_=*p*_*i*+1_/*ω*_*i*+1,*v*_^3^=(*B*_*i*+1_/*A*_*i*+1_)^3^*p*_*i*+1_
*a*_*i*+1_ − *a*_*i*_=*p*_*i*+1_(*B*_*i*+1_/*A*_*i*+1_)^3^ − *p*_*i*_(*B*_*i*_/*A*_*i*_)^3^=*p*_*i*+1_(*B*_*i*_/*A*_*i*_)^3^(*p*_*i*+1_ − 1/*p*_*i*+1_)^3^ − *p*_*i*_(*B*_*i*_/*A*_*i*_)^3^
=(*B*_*i*_/*A*_*i*_)^3^(*p*_*i*+1_ · (*p*_*i*+1_ − 1/*p*_*i*+1_)^3^ − *p*_*i*_)
(*p*_*i*+1_ − 1)^3^/*p*_*i*+1_^2^=*p*_*i*+1_^3^ − 3*p*_*i*+1_^2^+3*p*_*i*+1_ − 1/*p*_*i*+1_^2^=*p*_*i*+1_ − 3+3/*p*_*i*+1_
∴*p*_*i*+1_=*p*_*i*_+*ω*_*i*,*v*_, *ω*_*i*,*v*_ > 3
  (28) $$\begin{array}{c} {∴p_{i+1}\cdot \left(\frac{p_{i+1}-1}{p_{i+1}}\right)}^{3}-p_{i}=p_{i+1}-3+\frac{2}{p_{i+1}}-p_{i}=\omega_{i,v}-3+\frac{2}{p_{i+1}}>0\text{.} \end{array}$$
(2) ∴(*B*_*i*_/*A*_*i*_)^3^ > 0,∴*a*_*i*+1_ − *a*_*i*_ > 0,∴*a*_*i*+1_ > *a*_*i*_∴*p*_*i*+1_(*B*_*i*+1_/*A*_*i*+1_)^3^ > *p*_*i*_(*B*_*i*_/*A*_*i*_)^3^.
(3) $∴\underset{i⟶\infty }{\lim p_{i}/\omega_{i,v}^{3}}=\infty$,∵*ω*_*i*,*v*_^3^ > *ω*_*i*,*v*_^2^ > *ω*_*i*,*v*_,∴$\lim_{i⟶\infty }p_{i}/\omega_{i,v}^{2}=\infty$$\lim_{i⟶\infty }p_{i}/\omega_{i,v}=\infty$.

**Lemma 2 .**

The number *δ*_*γ*,*β*_^(!*s*!−*t*)^ of combination *u*_*γ*_^(!*s*!−*t*)^, if *p*_*γ*_^2^ < *β* < *p*_*γ*+1_^2^, *ω*_*n*,*v*_*p*_*n*_=*β*, then in the *∞* range, *δ*_*γ*,*β*_^(!*s*!−*t*)^=*p*_*n*_/*ω*_*n*,*v*_.

Prove:

(1) The number *u*(!*s*)/*i* of combination *δ*_*i*,*β*_^(!*s*)^.
For the convenience of narration, a is the number of *u*(!*s*)/*i* in the interval (0, *s*), B is the number of *u*(!*s*)/*i* in the interval (*s*, *β*), c is the number of *u*_*i*_^(!0)^ in the interval (−*s*, 0), and *D* is the number of *u*_*i*_^(!0)^ in the interval (0, *β* − *s*).
∵*s* < *p*_*γ*_, According to closed lemma 2, *E*=1.
According to the principle of symmetry, *C*=*E*=1, ∴*D*+*C*=*D*+*E*=*D*+1 .
According to the corresponding principle, *A*=*C*, *B*=*D* is obtained.
  (29) $$\begin{array}{c} ∴\;A+B=C+D=D+1-j\text{.} \end{array}$$
According to the above definition.
*δ*_*γ*,*β*−*s*_^(!0)^=*D* , *δ*_*γ*,*β*_^(!*S*)^=*A*+*B*=*C*+*D*=*D*+1=*δ*_*γ*,*β*−*S*_^(!*S*)^+1
(2) Number *δ*_*γ*,*β*_^(!−*t*)^ of combination *u*_*γ*_^(!−*t*)^.
Similarly.
∵*t* < *p*_*γ*_, According to closed lemma 2, *C*=1.
According to the correspondence principle, *B*=*D*
  (30) $$\begin{array}{c} \delta_{\gamma ,\beta +T}^{\left(!0\right)}=D+C\text{.} \end{array}$$
(3) The number of nontwo kinds of remainder combination is infinite.
∵*S* and *T* are constants and *β* is variable,
  (31) $$\begin{array}{c} \lim_{\beta ⟶\infty }\left(\beta -s\right)=\beta \lim_{\beta ⟶\infty }\left(\beta +t\right)=\beta \text{.} \end{array}$$
∴In the range of infinity,
  (32) $$\begin{array}{c} \delta_{\gamma ,\beta }^{\left(!s\right)}=\delta_{\gamma ,\beta }^{\left(!0\right)}\delta_{\gamma ,\beta }^{\left(!-t\right)}=\delta_{\gamma ,\beta }^{\left(!0\right)}\text{.} \end{array}$$
When,
  (33) $$\begin{array}{c} \omega_{n,v}p_{n}=\beta \delta_{\gamma ,\beta }^{\left(!0\right)}=p_{n}\delta_{\gamma ,\beta }^{\left(!s!-t\right)}=\frac{p_{n}}{\beta }\cdot \frac{p_{n}}{\beta }\cdot \beta =\frac{p_{n}}{\omega_{n,v}}\text{.} \end{array}$$
∵$\lim_{\gamma ⟶\infty }p_{n}/\omega_{n,v}=\infty$,∴$\lim_{\gamma ⟶\infty }\delta_{\gamma ,\beta }^{\left(!s!-t\right)}=\infty$.
That is to say, in the range of *∞*, the number of all nontwo types of remainder combinations is infinite.

The proof of lemma is finished.

### 3.3. Convergence of Mesh Discretization Method

Different results can be obtained under the grid settings, with large differences. If these results are in good agreement, it indicates that the simulation results are stable and reliable. On this basis, the convergence of the three-step two-layer grid discretization method is analyzed.

Order $\hat{p}n/h=\tilde{p}n/h+\bar{p}n/H,$$\hat{t}n/h=\tilde{t}n/h+\bar{t}n/H,$$\hat{\varphi }n/h=\tilde{\varphi }n/h+\bar{\varphi }n/H$

It can get the following equation:

(34) $$\begin{array}{c} \left(\frac{a^{n}-a^{n-1}}{\Delta t},w_{h}\right)+\left(\nabla \times \left(\prod_{h}\varphi^{n}-\varphi_{h}^{n}\right),w_{h}\right)=\left(E+G,w_{h}\right)\left(\beta^{n},v_{h}\right)=\left(a^{n},\nabla \times v_{h}\right)\left(\gamma^{n},v_{h}\right)=\left(\prod_{h}\varphi^{n}-\varphi^{n},v_{h}\right)+\left(K\left(p^{n}\right)t^{n},v^{h}\right)-\left(T_{4},v_{h}\right)\text{.} \end{array}$$

From the above theorem analysis and convergence analysis, it can be seen that, as long as the step *H* of the coarse space selected in the above algorithm satisfies *H*=*O*(*h*_3*k*+1_^*k*+1^), the two-layer mesh method established can maintain the optimal approximation of the solution of the mixed finite element method. The idea is applied to the reaction diffusion problem when the reaction term is a pressure *p*-related term (*f*(*p*, ∇*p*)), and the tensor *K* is a nonlinear term related to *p*. That is *K*(*p*). That is, to solve the original complex problem on the coarse mesh, and then to carry out Newton iteration on the fine mesh, that is to say, to solve a relatively simple problem on the fine mesh using the extended mixed finite element method, and then to correct it on the coarse mesh. By convergence analysis, the method can keep the best approximation of the mixed finite element solution.

## 4. Conclusions

NPE has important use value in many fields, such as science and technology manufacturing. But NPE usually contains many variables and is difficult to solve. NPE is an important branch of mathematics. It is one of the important tools needed to solve the linear algebraic discrete method of multivariable in reality. Based on the existing research results for NPE, the multivariable linear algebraic discrete method for NPE is studied. First, the NPE is discretized, the algebraic form of the nonlinear equation is transformed into the vector form, and the RS and IE are constructed to allocate the weights of different variable attributes. According to the given variable attribute weight, the multiple variables in the equation are discretized by linear algebra. Through the above steps, the research on the multivariable linear algebraic discretization method of NPE is completed in order to provide some help for the research in this field.

## Figures and Tables

**Table 1:** Value scale of *r*_*ij*_.

*r*_*ij*_	Definition	Explanation
1	Equal importance	Bothe the indicators of same importance
3	Slightly more important	One of the indicators is little more important
5	Obviously important	One of the indicators is clearly important
7	Much more important	One of the indicators is much more important
9	Extremely important	One of the indicators is extremely important
2468	Between the adjacent judgment	The discount degree of the above two judgments
1/2, 1/4, 1/6, 1/8	Inverse comparison	Inversely compare the two indicators

**Table 2:** The mean random consistency index.

Order number	1	2	3	4	5	6	7	8	9	10	11	12
*RI*	0	0	0.58	0.9	1.12	1.24	1.32	1.41	1.45	1.49	1.52	1.54

## Data Availability

The data used to support the findings of this study are available from the author upon request (zuoli@mjc-edu cn).

## References

[1] Sedeeg A. K. H., Nuruddeen R. I., Gómez-Aguilar J. F. (2019). Generalized optical soliton solutions to the (3+1)-dimensional resonant nonlinear Schrödinger equation with Kerr and parabolic law nonlinearities. *Optical and Quantum Electronics*.

[2] Kaur M., Kadam S., Hannoon N. (2022). Multi-level parallel scheduling of dependent-tasks using graph-partitioning and hybrid approaches over edge-cloud. *Soft Computing*.

[3] Ricarte G., Teymurazyan R., Urbano J. M. (2019). Singularly perturbed fully nonlinear parabolic problems and their asymptotic free boundaries. *Revista Matemática Iberoamericana*.

[4] Cheng J. Z., Jin L. Y., FangS M. (2020). Properties of solutions for a class of nonlinear pseudo parabolic equations. *Advances in Applied Mathematics*.

[5] Eichenberg C. (2018). Special solutions to a nonlinear coarsening model with local interactions. *Journal of Nonlinear Science*.

[6] Nishino T., Yokota T. (2019). Effect of nonlinear diffusion on a lower bound for the blow-up time in a fully parabolic chemotaxis system. *Journal of Mathematical Analysis and Applications*.

[7] Castro A. J., Rodriguez-Lopez S., Staubach W. (2017). L²-solvability of the Dirichlet, Neumann and regularity problems for parabolic equations with time-independent Hölder-continuous coefficients. *Transactions of the American Mathematical Society*.

[8] Kumar M., Tiwari A. K. (2018). Some group-invariant solutions of potential Kadomtsev-Petviashvili equation by using Lie symmetry approach. *Nonlinear Dynamics*.

[9] Wang Z. (2022). Inverse source problem of time dependent nonlinear parabolic equation. *Journal of Lanzhou University of Arts and Science(Natural Sciences)*.

[10] Liu H., Zhang R. P., Huo J. R. (2020). Finite difference method based on fast sine discrete transformation for solving semi linear parabolic equation. *Advances in Applied Mathematics*.

[11] Kaur M., Kadam S. S. (2017). Discovery of resources using MADM approaches for parallel and distributed computing. *Engineering Science and Technology, an International Journal*.

[12] Kevorkyants S. S. (2018). One solution of the forward problem of dc resistivity well logging by the method of volume integral equations with allowance for induced polarization. *Izvestiya - Physics of the Solid Earth*.

[13] Zhang T. Q. A. C., Zhang C. J. (2019). A general class of one-step approximation for index-1 stochastic delay-differential-algebraic equations. *Journal of Computational Mathematics*.

[14] Ouyang B. P., Li Y. F. (2020). Study on blow up of solutions of a class of Parabolic Equations with nonlinear gradient term. *Journal of Chongqing Normal University(Natural Science)*.

[15] Martens B., Gerdts M. (2019). Convergence analysis of the implicit euler-discretization and sufficient conditions for optimal control problems subject to index-one differential-algebraic equations. *Set-Valued and Variational Analysis*.

[16] Jadhav A., Kaur M., Akter F. (2022). Evolution of software development effort and cost estimation techniques: five decades study using automated text mining approach. *Mathematical Problems in Engineering*.

[17] Anguas L. M., Bueno M. I., Dopico F. M. (2019). A comparison of eigenvalue condition numbers for matrix polynomials. *Linear Algebra and Its Applications*.

[18] Kaur M., Jadhav A., Akter F. (2022). Resource Selection from Edge-Cloud for IIoT and Blockchain-Based Applications in Industry 4.0/5.0. *Security and Communication Networks*.

[19] Lu H. Q., Zhang Z. C. (2019). Blowup time estimates for a parabolic p-Laplacian equation with nonlinear gradient terms. *Zeitschrift für Angewandte Mathematik und Physik*.

